# Safety, Feasibility, and Tolerability of Ten Days of At-Home, Remotely Supervised tDCS During Gamified Attention Training in Children with Acquired Brain Injury: An Open-Label, Dose-Controlled Pilot Trial

**DOI:** 10.3390/brainsci15060561

**Published:** 2025-05-24

**Authors:** Athena Stein, Justin Riddle, Kevin A. Caulfield, Paul E. Dux, Maximilian A. Friehs, Philipp A. Schroeder, Michael P. Craven, Madeleine J. Groom, Kartik K. Iyer, Karen M. Barlow

**Affiliations:** 1 Acquired Brain Injury in Children Research Program, Child Health Research Centre, Faculty of Medicine, The University of Queensland, Brisbane, QLD 4101, Australia; 2 Carolina Center for Neurostimulation, Department of Psychiatry, University of North Carolina at Chapel Hill, Chapel Hill, NC 27516, USA; 3 Department of Psychology, Florida State University, Tallahassee, FL 32306, USA; 4 Brain Stimulation Division, Medical University of South Carolina, Charleston, SC 29425, USA; 5 School of Psychology, The University of Queensland, Brisbane, QLD 4072, Australia; 6 Psychology of Conflict Risk and Safety, University of Twente, 7500 AE Enschede, The Netherlands; 7 School of Psychology, University College Dublin, D04 C1P1 Dublin, Ireland; 8 Lise-Meitner Research Group Cognition and Plasticity, Max Planck Institute for Human Cognitive and Brain Sciences, 04103 Leipzig, Germany; 9 Department of Psychology, University of Tübingen, 72076 Tübingen, Germany; 10 German Center for Mental Health (DZPG), 72076 Tübingen, Germany; 11 NIHR MindTech MedTech Co-Operative, Institute of Mental Health, University of Nottingham, Nottingham NG7 2TU, UK; 12 Human Factors Research Group, Faculty of Engineering, University of Nottingham, Nottingham NG7 2RD, UK; 13 Academic Unit of Mental Health & Clinical Neurosciences, School of Medicine, Institute of Mental Health, University of Nottingham, Nottingham NG7 2TU, UK; 14 Brain Modelling Group, QIMR Berghofer Medical Research Institute, Herston, QLD 4006, Australia; 15 Queensland Pediatric Rehabilitation Service, Queensland Children’s Hospital, Brisbane, QLD 4101, Australia

**Keywords:** neuromodulation, TBI, pediatric, connectivity, HD-EEG, tDCS, at-home

## Abstract

**Background/Objectives:** Chronic attention problems occur in approximately 25% of children after acquired brain injury (ABI). When delivered daily, transcranial direct current stimulation (tDCS) may improve attention; however, access to daily in-clinic tDCS treatment can be limited by other commitments, including concurrent therapy, school commitments, and caregiver schedules. Treatment access can be improved through home-based interventions, though these require several practical and safety considerations in a pediatric ABI population. This study evaluated the safety, feasibility, and tolerability of remotely monitored at-home tDCS during online gamified attention training in pediatric ABI. **Methods**: We conducted a randomized, single-blind, dose-controlled clinical trial of at home tDCS in Brisbane, Australia (10 tDCS sessions; 20 min; 1 mA or 2 mA; bilateral dorsolateral prefrontal cortex). Participants attended our clinic at baseline for clinical assessments, fitting of the personalized tDCS headband, and training in how to use tDCS at home. All sessions were remotely supervised using live videoconferencing. We assessed the feasibility and tolerability of at-home tDCS and our customized, personalized at-home tDCS headband as primary outcomes. As secondary outcomes, we evaluated changes in functional connectivity (fc) and reaction time (RT). **Results:** Seventy-three participants were contacted over six months (January-June 2023) and ten were enrolled (5 males; mean age: 12.10 y [SD: 2.9]), satisfying a priori recruitment timelines (CONSORT reporting). All families successfully set up tDCS and completed attention training with excellent protocol adherence. There were no serious adverse events over the 100 total sessions. Nine participants completed all stimulation sessions (1 mA: *n* = 5, 2 mA: *n* = 4). Participants in the 2 mA group reported greater tingling, itching, and discomfort (all *p* < 0.05). One participant in the 1 mA group was unable to complete all sessions due to tolerability challenges; however, these challenges were resolved in the second half of the intervention by gradually increasing the stimulation duration across the 10 days alongside additional coaching and support. **Conclusions**: Overall, daily remotely supervised at-home tDCS in patients with pediatric ABI is safe, feasible, and tolerable. Our results support larger, sham-controlled efficacy trials and provide a foundation for the development of safe and effective at-home stimulation therapeutics that may offer targeted improvement of neurocognitive symptoms in children.

## 1. Introduction

Attention problems following pediatric acquired brain injury (ABI) are the most common ongoing complaint, affecting up to 25% of children [1,2]. These attention problems significantly impact daily living, educational attainment, and community participation; yet pharmacotherapy is often poorly tolerated, and compliance is low [2,3,4,5]. Acquired brain injury is a leading cause of death and lifelong disability [6,7], especially when acquired in childhood; and its sequelae are multifaceted, often posing a lifelong burden affecting participation in education, the workforce, and communities [4]. Pharmacological therapy is the gold-standard treatment for attention problems [8]. However, side effects such as weight loss, sleep disturbance, and stigmatization decrease adherence to medication [3,9], and prescribing restrictions have decreased access in the community.

Non-invasive brain stimulation (NIBS) such as transcranial direct current stimulation (tDCS) offers a potential alternative treatment for attention problems [10,11] following pediatric ABI [12,13]. tDCS uses low-intensity currents to transiently alter membrane excitability and increase the potential for neuroplastic change on a network level [14,15]. Previous research has shown promising results for tDCS in adult ABI populations in terms of attention and brain network changes [16,17]. However, the effects of tDCS vary across patients due to many inter-individual factors, including differences in structural and functional network organization, particularly in the executive control (ECN), salience (SN), and default mode networks (DMN) [13,16,18,19,20,21,22,23,24]. High-density electroencephalography (HD-EEG) is emerging as a promising, portable tool to measure functional connectivity (fc) and account for brain- and injury-related differences in tDCS response across heterogeneous populations, with the added advantage of being more child-friendly and cost-effective than traditional magnetic resonance imaging (MRI) [25,26,27].

Repeated tDCS sessions with concurrent cognitive training are required to produce robust and prolonged effects, potentially mitigating some of the inter-individual variability in response [28], as demonstrated in both healthy and clinical populations [16,17,18,29,30]. Improvements in attention following adult traumatic brain injury (TBI) have been reported following 10 to 15 daily sessions [16,17]. However, conducting consecutive sessions of tDCS in a clinical environment can be challenging, with drop-out rates of up to 40%, especially in childhood populations, due to the impact on school, work, and family life, as well as concurrent ongoing medical and rehabilitation-related commitments [31].

Delivering tDCS at home alleviates some of these challenges in adults by increasing convenience and decreasing participant burden [31,32,33]; yet few studies have demonstrated the safety, feasibility, and methodology in clinical populations, with a particular scarcity in pediatric populations [13,34]. To address this knowledge gap, we designed the first interventional clinical trial investigating at-home tDCS during gamified attention training with special considerations for safety, adherence to protocol, and data quality in pediatric ABI [35]. We examined the safety, feasibility, and tolerability of a 10-day at-home tDCS intervention delivered at 1 mA or 2 mA in children with ABI. As secondary outcomes, we also assessed cognitive, neurophysiological, and behavioral measures of attention to provide a rationale for future, larger-scale efficacy studies. Our study presents a novel application of daily at-home, remotely supervised tDCS in a vulnerable clinical population. Augmented by neuroimaging and current modeling techniques, our trial provides feasibility and preliminary efficacy data to inform an individualized treatment framework in larger clinical trials.

## 2. Materials and Methods

### 2.1. Study Design

**Figure 1:**
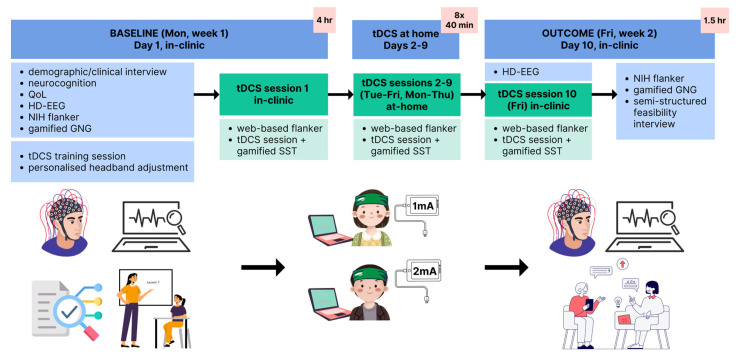
Baseline assessments, setup instructions, and primary outcomes were performed in clinic, after which families conducted tDCS sessions (randomized to 1 mA or 2 mA) at home with remote supervision. Follow-up sessions were conducted at 1 week and 4 weeks by telehealth. QoL, quality of life; HD-EEG, high-density electroencephalography; tDCS, transcranial direct current stimulation; SST, stop signal task; GNG, go/no-go task; NIH, National Institutes of Health.

This feasibility study used a randomized, parallel, two-arm, dose-controlled, open-label, single-blind clinical trial design (Figure 1). The study was conducted between January and August 2023. Ten participants received ten sessions each of tDCS (randomized to 1 mA or 2 mA doses) during gamified training over two weeks. Baseline neurobehavioral assessments were conducted at the Centre for Children’s Health Research, Queensland Children’s Hospital (Brisbane, Australia), together with participant/caregiver at-home tDCS training. Intervention sessions 1 and 10 took place in the clinic and sessions 2–9 at home. Reaction time was measured daily, prior to stimulation. Neurophysiological brain activity changes were measured in clinic using HD-EEG (Day 1 and Day 10). To estimate the cortical tDCS ‘dose’ received, we simulated tDCS cortical E-field models using the participant’s structural MRI (optional assessment) (see Section 2.9, below). Follow-up clinical assessments occurred by telehealth 1 and 4 weeks later. Participants were asked to maintain their regular medication schedule without change for the period of the study. The trial was conducted in accordance with Good Clinical Practice, and ethical approval was received from the Children’s Health Queensland and University of Queensland Human Research Ethics Committees (HREC/21/QCHQ/73034). All patients and parents/guardians gave written informed assent or consent, respectively, to study participation and publications. The trial was registered on the Australian New Zealand Clinical Trials Registry: ACTRN12622001562763.

### 2.2. Inclusion and Exclusion Criteria

Participants were recruited using flyers and direct email/telephone contact using an existing Queensland Pediatric Rehabilitation Service database where families had consented to research-related contact. Participants were eligible if they were aged 7–18 years (inclusive), had sufficient English language skills to participate, and were at least three months post-injury (mild TBI) or one-year post-injury (moderate/severe TBI and ABI, where these cut-offs were determined as the period of injury stabilization). ABI was defined as any injury to the brain occurring after birth, such as TBI, stroke, or infection [6]. TBI injury severity was defined according to the American Congress of Rehabilitation Medicine Criteria [36] and as detailed in our previous work [37].

The exclusion criteria were as follows: absence of home internet access; contra-indications to tDCS (Supplementary Materials); hypoxic–ischemic encephalopathy [38]; past medical or psychiatric history that could influence tolerability or performance; profound memory impairments on neuropsychological testing; recent or planned change in neuroactive drugs (i.e., anticonvulsants, benzodiazepines, GABA antagonists, etc.); pre-existing neurological disorders; inability to perform cognitive tasks; or presence of a condition or abnormality that in the opinion of the investigator would compromise the safety of the patient or the quality of the data.

### 2.3. Sample Size, Randomization, and Blinding

The sample size (*n* = 10) was determined a priori to allow reasonable estimation of feasibility and safety, similar to previous studies in stroke populations [39,40,41]. The randomization sequence was computer-generated using a block size of 4. Participants and families only were blinded to group allocation.

### 2.4. Primary Outcomes

#### Safety, Feasibility, and Tolerability

The feasibility of the trial was reported according to recognized guidelines [42], including the Consolidated Standards of Reporting Trials (CONSORT) statement extension to randomized pilot and feasibility studies checklist [43]. Feasibility measures included participant recruitment and retention and protocol acceptability and adherence. An a priori retention target was defined at 80% (of sessions completed and participants retained). Unstructured interviews were used to inform about intervention acceptability and adherence, including the personalized tDCS headband, and online gamified attention training. Safety and tolerability were assessed using a formal structured interview.

### 2.5. Secondary Outcome Measures

As a proxy for attention, we measured change in flanker [44,45] RT and go/no-go [46,47,48] RT across the intervention. We measured change in flanker RT before and after the 10 days of tDCS using the NIH Toolbox (iPad; Toolbox Assessments, Inc., Chicago, IL, USA) [49], which was placed on a table in front of the seated participant and completed with the index finger of the participant’s dominant hand. This 3 min task consists of 24 congruent and incongruent trials and has strong validity and test–retest reliability in pediatric TBI populations [50]. Flanker RT was also measured daily immediately prior to intervention sessions using a web-based flanker task [45] involving 80 trials (40 trials × 2 blocks) of congruent or incongruent flanker stimuli. Participants engaged in the task on a laptop while seated at a desk, using two fingers on their dominant hand to press the left and right arrow keys to respond. This was repeated at 1- and 4-week follow-ups. The ‘Wormy Fruit’ go/no-go task [46,47,48] RT was also used to assess attention at baseline, Day 10, 1 week, and 4 weeks following the intervention, involving presentation of successive images of a whole apple (‘go’ stimulus) or a ‘wormy apple’ (‘no-go’ stimulus) (150 trials; 75% go, 25% no-go; 200 ms presentation time). Participants engaged in the task on a laptop while seated at a desk, pressing the spacebar with their dominant hand to respond. High-density EEG was measured at baseline and following the tDCS intervention (further detail below) to assess brain network changes following tDCS across the wide range of injury severities. Behavioral attention was measured using Conner’s 4 short-form parent report (Pearson Assessments, San Antonio, TX, USA).

### 2.6. Intervention

For the intervention, 1 mA or 2 mA tDCS (anode over left dlPFC, cathode over right dlPFC) was applied for 20 min during gamified attention training tasks daily for 10 consecutive weekdays (see Figure 1). Two dosage arms were incorporated to expand feasibility and tolerability data in this population, given that tDCS is often used at intensities up to 2 mA. Stimulation was given for 20 min using 5 × 5 cm saline-soaked sponges (NeuroConn DC Stimulator Mobile, Ilmenau, Germany; see device information in the Supplementary Materials). The anodal stimulation site was the left dlPFC (F3 on the 10/20 EEG system), and the cathode was placed contralaterally on the right dlPFC (F4). The dlPFC was chosen as a target due to its central role in executive dysfunction following ABI [24]. The anode was specifically placed on the left dlPFC informed by previous evidence demonstrating cognitive and EEG power changes following tDCS in adult ABI [16]. The scalp current density for each electrode was calculated at 0.04 mA/cm^2^ and 0.08 mA/cm^2^ for the 1 mA and 2 mA dosages, respectively. The ramp-up and ramp-down time was 30 s. The online attention training involved two gamified adaptive stop signal tasks (SSTs), which alternated each day to increase engagement: (A) the ‘Fairy Game’ stop signal task [51] and (B) the ‘Sorting Game’ stop signal task [52] (Supplementary Materials). Online training was randomized to one of two task orders (ABAB or BABA) across participants. SST training began immediately upon tDCS ramp-up and continued for 17–19 min.

### 2.7. Home-Based Protocol

Participant and caregiver training involved tDCS education, tDCS headband application, a live demonstration, and ‘hands-on’ practice. The parent/caregiver and participant were required to show confident, independent use of tDCS and setup of training task equipment before using tDCS at home. Ease of tDCS setup was facilitated by a personalized headband using head circumference, a nasion marker, and F3/F4 placement (10–20 EEG system). A laptop (Acer Aspire 1, New Taipei City, Taiwan) was provided for daily remote supervision, attention assessments, and attention training (Supplementary Materials).

At-home sessions began with the study staff member (preceptor) connecting with the participant over Zoom (Zoom Communications; San Jose, CA, USA). All session activities were completed under video supervision. After the participant completed the daily flanker task, the preceptor would supervise the participant and/or caregiver to saturate the tDCS sponges with saline, attach the sponges to the appropriate Velcro dots on the headband (corresponding colors), and place the tDCS headband with sponges on the participant’s forehead, aligning the nasion marker to their nasion and removing hair from underneath the sponge electrodes. The preceptor then visually assessed the placement of the electrodes and provided feedback. Once the preceptor confirmed that the headband was placed correctly, the participant and/or caregiver was given permission to start the impedance check. If the impedance was satisfactory (defined as within 15 kΩ), permission was given to proceed with the session. The participant and/or caregiver then started the attention game and tDCS simultaneously, and the preceptor supervised the participant performing the attention game during tDCS for the entire duration of the session. The preceptor aimed to engage minimally with the participant, providing only light encouragement every 7–10 min and/or gentle reminders to pay attention if the participant became distracted. At the end of the session, the preceptor supervised the participant to correctly clean and pack away the tDCS device and confirmed the scheduling of the next session. The staff member recorded intervention details such as session date; time and location; recent medication; caffeine and food intake; presence of distracters in the environment; patient-reported tiredness; tDCS sensations, including tingling, itching, burning, pain, and fatigue; and changes in alertness, as well as associated severity (adapted from Fertonani et al. 2010 [53]), the effect of tDCS-associated sensations on the participants’ ability to concentrate on the game, and tDCS impedances.

Two additional intervention preceptors (graduate students) were trained to facilitate remote monitoring. Preceptor training involved education about tDCS, tDCS setup, self-experience of tDCS, supervision of a remote session, and finally precepting a remote session with supervision. Remote sessions were recorded and retrospectively reviewed by A.S. to evaluate safety, protocol compliance, and data quality.

### 2.8. Baseline Assessments

Baseline assessments included population-validated measures of cognitive function (CNS Vital Signs [CNSVS; Morrisville, NC, USA]) [54] and attention (Behavior Rating Inventory for Executive Functioning; BRIEF2 Parent Report, Second Edition; PAR Inc., Lutz, FL, USA). The Conner’s 4 Short form (parent version) [55] was used to assess behavioral attention at baseline and at the 4-week follow-up.

### 2.9. Electric Field Modeling

To estimate the cortical tDCS ‘dose’ received, we simulated cortical E-field models. Individual head models were created for ABI participants from T1 and T2 scans (*n* = 9; details in Supplementary Materials) using the simNIBS headreco segmentation and meshing pipeline (Copenhagen, Denmark) [56,57,58] in MATLAB (R2021a, The MathWorks, Inc., Natick, MA, USA). The left dlPFC/right dlPFC montage was modeled according to the F3-anode/F4-cathode (10–20 EEG; electrode size: 5 × 5 cm). The anodal and cathodal current intensity was +1 mA/−1 mA and +2 mA/−2 mA in the 1 mA and 2 mA conditions, respectively, and default tissue conductivity values were utilized. The mean E-field magnitude (E_norm, v/m) was calculated in the F3 and F4 regions of interest (further details provided in the Supplementary Materials).

### 2.10. HD-EEG Functional Connectivity

A 128 channel cap (Geodesic EGI Hydrocel GSN 130; Electrical Geodesics, Inc., Eugene, OR, USA) was used to record 5 min of resting eyes open (EO) while watching the resting-state video, ‘Inscapes’ [59], 5 min of resting with eyes closed (EC), and 3 min of task-based (TB) EEG, sampled at 1000 Hz using Net Station (Version 5.4.2; Philips Electrical Geodesics, Eugene, OR, USA) (See Supplementary Materials).

Source-based high-density encephalography (HD-EEG) was used to examine resting and task-related source-derived fc changes following the intervention using amplitude envelope correlation. The Brainstorm toolbox (Version February 2023) [60] was used for source-based connectivity analysis. Where available, T1 MRI scans were used for subject anatomy (9 ABI participants). An age-specific (10–14 years) asymmetric T1 template from the NIHPD database was used for one participant as MRI was not available [61,62].

Mean spectral power was computed via Welch’s estimate [63] and extracted for all channels, as well as in a subgroup of a frontal division, temporal division, and parieto-occipital division of channels [64]. T1 scans were segmented using CAT12 [65], and EEG channel files were then co-registered. Boundary element model (BEM) [66] surfaces were generated for each participant. Diagonal noise covariance was computed from the Brainstorm identity matrix, and a head model was created using 15,002 vertex segmentations with adjoint formulation. Forward model source reconstruction was run using dynamic Statistical Parametric Mapping (dSPM) [67] with a single kernel. Mean connectivity values for each participant were extracted from source-reconstructed time series across 68 cortical regions (Desikan–Killiany [68] atlas) for all epochs. Orthogonalized envelope correlation was conducted using a Hilbert time–frequency transformation [69] across delta, theta, alpha, beta, gamma, and broadband frequencies using static time resolution with a sliding window length of 1500 ms and a 50% overlap.

Significant group differences in subnetwork connectivity were identified using network-based statistics (NBS) [70]. Region-of-interest-based analyses were also conducted within the DMN, SN, and ECN as important attention-related networks (Supplementary Materials) [71].

### 2.11. Statistical Analysis

Statistical analysis and data visualization were performed using MATLAB Version 9.12 (R2022a, The MathWorks Inc., Natick, MA, USA), RStudio (Version 2023.9.1.494, PBC, Boston, MA, USA), the Just Another Statistics Program (JASP; Version 0.16.2.0 for Windows), and GraphPad Prism Version 9.0.0 (GraphPad Software, Boston, MA, USA).

Flanker, stop signal task, and go/no-go reaction times in each group were described (see Supplementary Tables S1–S3). To investigate changes in functional connectivity following the intervention, repeated-measures ANOVA was conducted using NBS [70], where all participants were grouped together regardless of dose due to the small sample size. Tests were performed at the group level, with age as a covariate at a threshold of F = 9.61 with exchange blocks (for paired comparisons) and 5000 permutations at *p* = 0.05. Mean connectivity values for each participant were extracted from any identified significant subnetworks. To define the networks identified in the whole-brain analysis, we conducted network of interest analyses by creating network ‘regions of interest’ (ROIs). For the ROI analysis, two-tailed *t*-tests were conducted to investigate changes in connectivity (DMN, ECN, and SN) over time across each frequency. Given the exploratory nature of these analyses, multiple-comparison corrections were not performed. Correlation analysis was conducted to examine the association between baseline fc and RT, as well as RT change and fc change, and RT change and E-fields (Supplementary Materials).

## 3. Results

### 3.1. Participants

**Figure 2:**
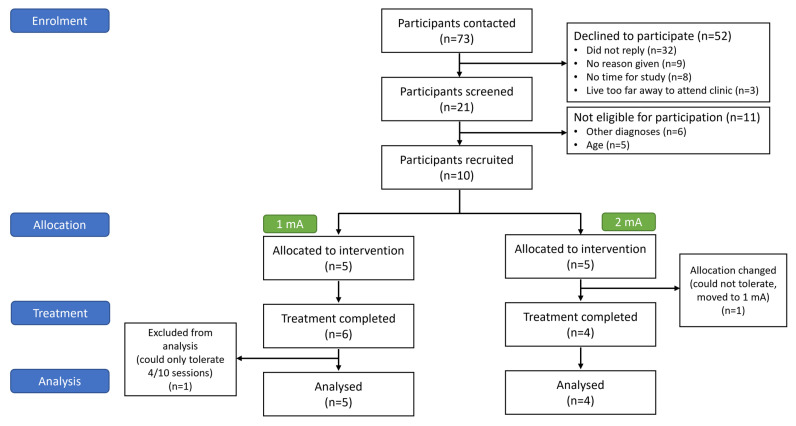
CONSORT flow diagram.

Seventy-three participants consented to being contacted about the study. Ten participants (mean age: 12.1 y [SD: 2.9 y], 5 [50%] males) at 4.2 years (SD: 3.8 y) post-injury were enrolled. All participants had TBI: mild (*n* = 6) or severe (*n* = 4). Demographic and clinical details are reported in Table 1 and Table 2. In total, 100 tDCS sessions were conducted (see Figure 2).

### 3.2. Feasibility and Tolerability

#### 3.2.1. Feasibility

Other than participants not replying, the main reasons for non-participation in the trial were lack of time (*n* = 8) and distance to travel (*n* = 3). Eleven participants met exclusion criteria (comorbid diagnoses (*n* = 6) and incorrect age (*n* = 5)), resulting in a recruitment rate of 13.7% (1.7 participants per month). Of 100 at-home tDCS sessions, 93 were supervised by A.S. and 7 by the trained preceptors. Preceptor training took approximately four hours. All sessions correctly adhered to protocol procedures on retrospective video review.

Participants did not report any problems with the setup, tDCS application, accessing remote supervision, or completion of the web- and software-based attention assessments and attention tasks at home. After the initial at-home session, all children over the age of 10 years preferred to conduct sessions mostly independently (with little help from their guardian, although remote supervision by study staff was continued throughout). Five participants (50%) conducted the intervention across several home environments. In all cases, a quiet, distraction-free setting was established. The intervention was conducted after school in seven participants and before school in three participants. The web-based Sorting SST was affected by local internet speed, resulting in variations in stimulus presentation times. Each participant played five Sorting SST sessions and five Fairy SST sessions, except one participant who played only the Fairy SST for all ten sessions due to web-based technical difficulties. Based on SST performance, participants remained engaged in the tasks throughout the sessions (Supplementary Tables S2 and S3).

#### 3.2.2. Tolerability

**Figure 3:**
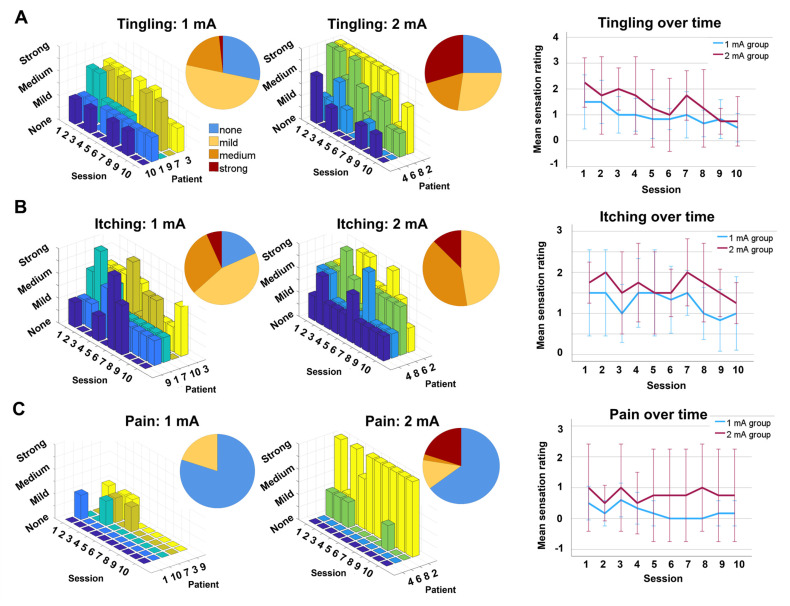
Participant ratings of tingling (**A**), itching (**B**), and pain (**C**) sensations during tDCS sessions across groups. Individual ratings over time are demonstrated in 3D bar graphs, and the group proportions are shown in the pie charts (none = blue, mild = yellow, medium = orange, strong = red). The average sensation ratings (1 = mild, 2 = medium, 3 = strong) are demonstrated in the line graphs over the 10 sessions of tDCS, and error bars represent 1 standard deviation from the mean. Bar and chart colors provide arbitrary distinctions between data elements. Nine participants included. mA, milliamp.

All intervention sessions took place. One participant (an 11-year-old male) was unable to tolerate 2 mA tDCS and was moved to the 1 mA group after the first session, which was tolerated well. Another participant (a 7-year-old male) was unable to tolerate 1 mA tDCS for more than 5 min initially. As he wished to remain in the trial, the tDCS duration was gradually increased over six sessions, allowing successful completion of the remaining four tDCS sessions for the full duration (excluded from analyses; see Supplementary Figure S1 for this participant‘s sensation ratings over time). The 2 mA group experienced more tingling (Fisher’s exact χ^2^ = 16.3, *p* < 0.001), itching (χ^2^ = 10.2, *p* = 0.02), and pain (χ^2^ = 15.0, *p* < 0.001) sensations; however, none became visibly distressed, and all wished to continue the session. The intensity of these sensations decreased over time (Figure 3). Mild skin redness was noted on the electrode sites following all sessions of tDCS. No serious adverse events were observed.

### 3.3. Qualitative Feedback

All participants and their families gave positive feedback about their participation, and all reported that a home-based study was feasible and preferable to a daily in-clinic study. Participants valued the telehealth supervision, reporting that this increased their confidence. All found the personalized headband easy to use. There were no tDCS- or attention task-related technical difficulties. The gamified cognitive training was well received, with each game favored equally.

### 3.4. Attention

**Figure 4:**
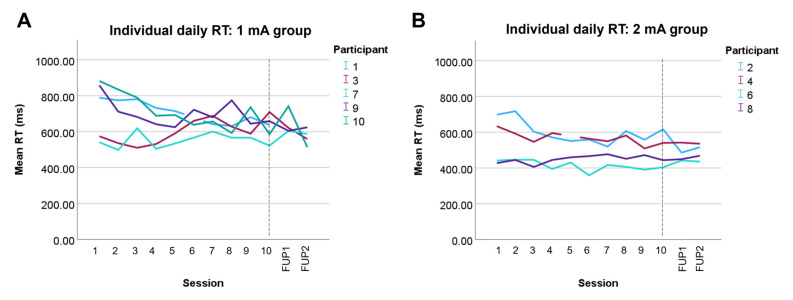
Individual trajectories of daily web-based flanker RT during intervention period and at 1- and 4-week follow-ups in (**A**) 1 mA group and (**B**) 2 mA group. Dotted lines signify the end of the 10-day intervention. RT, reaction time; mA, milliamps; ms, milliseconds.

Individual RTs are displayed in Table 2 and Figure 4. The mean baseline flanker RT was significantly higher (slower) in the 1 mA group compared to the 2 mA group (Table 2; t = 3.2, *p* = 0.01). Individual reaction times showed a visual (non-statistical) trend towards improvement over time across both dosages (Figure 4). There were no changes in behavioral attention.

**Table 2:** Individual patient demographic information and reaction time change.

ID	Age at Study Baseline (Years)	Time Post-ABI (Years)	Sex	Injury Severity	Medication with Effect on Cortical Excitability ^◇^	tDCS Dosage Assigned	tDCS Dosage Received	Pre-tDCS Flanker RT (ms)	Post-tDCS Flanker RT (ms)	RT Change Following Intervention
1	9.7	8.6	M	Severe	Non-stimulant ADHD medication (D)	1 mA	1 mA	766.29	637.54	Faster
3	16.5	0.3	F	Mild	Amitriptyline (D)	1 mA	1 mA	604.26	623.34	Slower
5	7.4	2.0	M	Mild	None	1 mA	1 mA *	853.74	924.45	Slower ^+^
7	12.5	10.0	M	Severe	SSRI (I) Stimulant (I)	1 mA	1 mA	545.41	422.13	Faster
9	9.4	0.8 ^++^	F	Severe	None	1 mA	1 mA	722.04	596.68	Faster
10	10.8	4.1	M	Mild	Atypical antipsychotic (I) Stimulant (I)	2 mA	1 mA **	856.20	685.61	Faster
2	11.9	9.6	F	Severe	None	2 mA	2 mA	484.59	382.13	Faster
4	15.8	2.0	F	Mild	Amitriptyline (D) Topiramate (D)	2 mA	2 mA	625.85	525.21	Faster
6	12.8	2.8	M	Mild	None	2 mA	2 mA	389.14	322.12	Faster
8	14.3	1.3	F	Mild	Stimulant (I)	2 mA	2 mA	361.25	383.45	Slower

**Figure 5:**
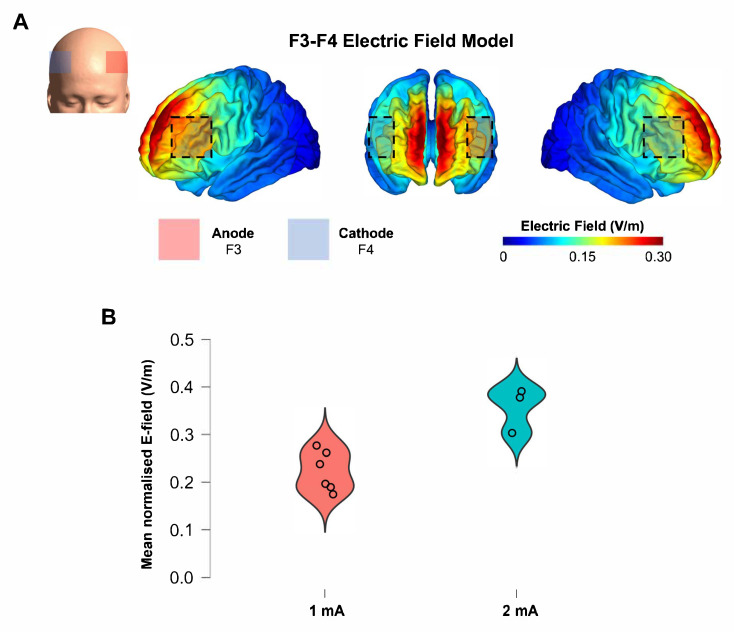
Mean normalized electric field (E-field; V/m) in 1 mA and 2 mA tDCS groups. (**A**) Visualization of E-field model with scalp electrode placement. Colors represent electric field on scale from 0 V/m (blue) to 0.3 V/m (red) (**B**) Mean normalized E-field in left dlPFC (anode) in 1 mA and 2 mA groups. tDCS, transcranial direct current stimulation; dlPFC, dorsolateral prefrontal cortex; E-field, electric field.

Patient ID corresponds to sensation ratings in Figure 4 and RT changes in Figure 5. tDCS, transcranial direct current stimulation; RT, reaction time; mA, milliamp; SSRI, selective serotonin reuptake inhibitor; ADHD, attention deficit hyperactivity disorder. ^◇^ Effect of medication on cortical excitability given in brackets: I, increase in cortical excitability; D, decrease in cortical excitability. * Session length ramped up from 5 min to 20 min (by 6th session) due to problems with tolerability. ** Pt could not tolerate assigned dosage (2 mA) and was moved to 1 mA group. ^+^ Participant excluded from RT analysis as 40% of tDCS sessions fully completed. ^++^ Participant with severe TBI was deemed to be stable and able to participate at 10 months post-injury according to clinical assessment by the principal investigator.

### 3.5. Simulated E-Fields

We computed E-field models for the nine participants with individual T1 and T2 scans (*n* = 6, 1 mA; *n* = 3, 2 mA). The normalized mean E-field strength under the anode (F3, left dlPFC) was significantly different between groups (t = 3.9, *p* = 0.006; 1 mA group: 0.22 (SD 0.04) V/m; 2 mA group: 0.36 (SD 0.05) V/m; Figure 5). Change in go RT (‘Wormy Fruit’ task) following the intervention was strongly correlated with E-fields under the anode (Pearson’s r = −0.73, *p* = 0.039). There were no significant correlations between flanker RT change and mean anodal E-field.

### 3.6. HD-EEG Functional Connectivity

**Figure 6:**
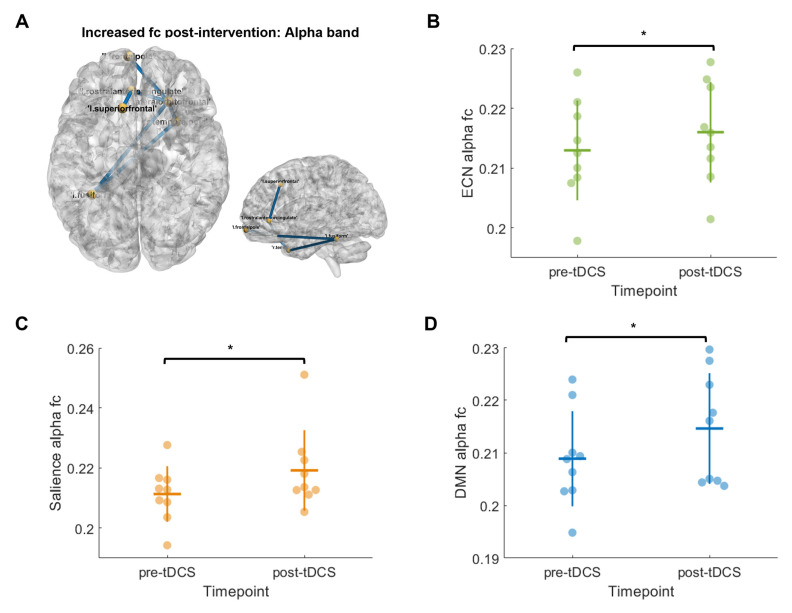
Significantly increased alpha-band functional connectivity following intervention seen in both whole-brain NBS analysis (eyes-closed condition) (**A**) and region-of-interest analyses (eyes-open condition): (**B**) executive control network, (**C**) salience network, and (**D**) default mode network (*n* = 9). Yellow spheres indicate nodes and blue lines indicate edges between notes. * indicates statistical significance at *p* < 0.05. ECN, executive control network; DMN, default mode network; fc, functional connectivity.

We analyzed overall changes in fc over time as an exploratory analysis (*n* = 9; Figure 6), using amplitude envelope correlation. Following the intervention, eyes-open (EO) resting-state theta, beta, and gamma fc and eyes-closed (EC) alpha and gamma fc significantly increased in several whole-brain NBS subnetworks (t = 3.1, *p* < 0.001, NBS; Figure 6, Supplementary Figure S2). Contrary to our hypotheses, task fc did not significantly change (*p* > 0.05). Changes in fc were not associated with flanker or go RT change (*p* > 0.05).

Using ROI analysis in pre-defined attention networks, we found significantly increased EO alpha connectivity in the DMN (t = 2.6, *p* = 0.03), ECN (t = 2.8, *p* = 0.02), and SN (t = 2.6, *p* = 0.03) following the intervention (Figure 6). This indicated that the alpha-band connectivity results seen in the whole-brain NBS analysis likely corresponded to alpha fc increases in the ECN, SN, and DMN specifically. There were no changes in spectral power over time. See Supplementary Table S4 for details of nodes in significant subnetworks.

## 4. Discussion

Repeated tDCS sessions are likely required for clinically meaningful effects; yet the optimal repetition interval of these sessions is not fully understood [30,72,73]. For tDCS to be considered as a treatment for attention problems following childhood brain injury, a feasible and safe at-home alternative is required to reduce the travel and commitment burden of tDCS in the clinic [35,74,75]. This trial provides crucial information that is needed to conduct at-home tDCS in non-neurotypical populations where research is scarce due to the associated additional safety concerns (e.g., atypical anatomy, altered cortical excitability, and comorbidities) and barriers to uptake (acceptability, parent/family concerns [12,75], and family burden) [19,76], especially when provided alongside traditional rehabilitation/therapies [77]. We demonstrated that 10 days of home-based, remotely supervised tDCS during attention training can be safe, feasible, and tolerable in children with acquired brain injury, providing evidence to inform larger trials evaluating the efficacy of tDCS.

Recruiting participants from a previously established recruitment registry in our research team was a significant strength of our study. In our experience, the previously built rapport between the research team and participants improved recruitment rates as well as participant retention. Further, over half of the participants had participated in our previous mechanistic trial involving a single session of tDCS [13], which meant that the majority of participants and their families were familiar with the use of tDCS. Recruiting naïve tDCS participants would likely post more challenges due to ongoing negative perceptions of the technique in the community [78]; therefore, establishing recruitment registries may be an important way to improve patient recruitment and retention.

By providing a personalized tDCS headband, computer equipment, and remote monitoring, we developed a protocol that was both feasible and acceptable. All sessions were attended, and there were no losses to follow-up. Families affirmed acceptability and their preference for an at-home program, and parents and children felt confident in administering the tDCS at home. Using special color-coded placement markers, pictorial instructions, and supervision, all teens preferred and managed to apply tDCS themselves, which was confirmed by remote supervision and device impedance information. Similar to Charvet et al. 2020 [35] and Cappon 2024 [33], all the necessary equipment to deliver and monitor the intervention was provided. Protocols aided consistent training and assessment involving both participants and their caregivers helped to ensure safe and correct tDCS use. It is important to ensure sufficient training for both participants and parents/guardians in at-home studies involving pediatric populations. Further, the portability and remote monitoring allowed participants to choose when and where sessions were conducted, fitting with school, homework, and family commitments. Overall, we developed a simple and fail-safe setup that allowed consistency across different environments that has potential for in-school use in the future.

Continued remote supervision throughout the trial was an important aspect of feasibility and compliance. In our study, all families commented on the importance of this daily remote supervision for their confidence. We also demonstrated the relative ease of remote supervisor training. After four hours of preceptor training, universal safe and correct protocol compliance was achieved.

Engagement in attention training during tDCS is essential, as previous literature has indicated that cognitive changes following tDCS are brain-state-dependent [79]. The gamification was well received by our participants, who reported the attention training to be enjoyable and believed that the game elements increased their engagement, perhaps reducing dropout. Remote supervision and the use of adaptive stop signal tasks in our study (i.e., tasks which became harder or easier in real time as the children played) likely contributed to the high levels of participant engagement seen throughout the intervention [80,81].

Our results significantly contribute to safety data for at-home tDCS during cognitive training, which has not been previously studied in pediatric TBI populations. No serious adverse events occurred, and skin sensations were well tolerated in most children. Similar to previous work in pediatric stroke, tingling and itching were the most commonly reported sensations [39,40,41,82]. We found that tolerability could be improved by decreasing the intensity and gradually increasing the duration of tDCS. Some participants habituated to tDCS-induced sensations over the intervention period. To improve tolerance to tDCS sensations, we suggest that future trials, especially in young children, should consider a gradual increase in stimulation duration and intensity over several days.

The good tolerability of tDCS at 2 mA is important when planning future trials. We found that greater improvement in reaction times was associated with higher E-field strengths. Several recent publications have proposed that differences in tDCS response are due to variability in E-fields generated at the cortex [22,83]. Supporting this, we found that left dlPFC E-field strength was associated with reaction time change, and that the estimated E-fields in our study (0.22 V/m and 0.36 V/m in the 1 mA and 2 mA groups, respectively) were similar to those reported in other tDCS studies where improvements in cognition were found [20,22] and higher than those in studies reporting E-fields associated with no cognitive changes [22]. Future research in this area is needed, however, as evidence has suggested a non-linear tDCS dose response in healthy populations, but little information is available in clinical samples [84,85,86,87].

As a feasibility study, there was insufficient power to draw any conclusions regarding the effect of tDCS on attention. Although reaction time showed a trend towards improvement over time in some participants, this may reflect the general effect of training. Nonetheless, a training effect over a short period of time would be interesting, given that children with ABI commonly have difficulty with executive [88], attention, and memory function [89]. Overall, future larger, sham-controlled trials are needed to determine the efficacy of tDCS effects on attention, with consideration of the number of treatment days and increased follow-up.

Previous research indicated that functional brain connectivity predicts changes in attention task performance following tDCS in pediatric ABI [13]. To understand how attention networks may be altered with tDCS, using HD-EEG, we investigated changes in fc across individual frequency bands following the intervention [90,91,92]. The most notable finding was increased alpha-band fc following the intervention, seen independently in both the whole-brain and region-of-interest analyses (which delineated these changes in the ECN, SN, and DMN specifically). These results mirror those seen in Ulam et al. 2015, who found increased alpha activity in adults with TBI following 10 days of tDCS, suggesting improved regulation of cortical excitability [16]. Although future validation in larger samples is required, we suggest that increased alpha connectivity may indicate a shift in cortical excitatory/inhibitory balance towards a less hyperactive state [93]. These results are particularly interesting in the context of the DMN, SN, and ECN, as changes in these networks following ABI has previously been suggested to relate to network dysfunction or increased recruitment of resources (i.e., compensatory network activity) [24,71,94,95,96,97]. Overall, we provide a framework to support future use of HD-EEG to measure fc in tDCS interventions involving children with ABI. Such neurophysiological markers of tDCS response are crucial given the variability in tDCS response, especially in clinical populations and children. Brain connectivity and source-based EEG should be further investigated as a child-friendly alternative to MRI.

### Study Limitations

The small sample size, the single blinding, and the lack of a sham stimulation condition limited the ability to draw conclusions regarding the efficacy of tDCS. Further, researcher familiarity with participants in the small research group could have created subconscious bias. Nevertheless, this relatively small feasibility study is important for conducting larger home-based tDCS studies following childhood ABI, and our feasibility results are similar to those seen in previous studies [39,40,41]. Although we monitored tolerability throughout the study using standardized post-session questionnaires, one participant retrospectively reported high levels of pain on the sensation questionnaire after completing sessions despite minimal discomfort being reported or obvious during stimulation. This discrepancy highlights the limitations of current self-report measures in pediatric populations. Future research should consider developing and validating child-specific rating tools that more accurately capture discomfort and sensory experiences during brain stimulation, particularly in real time, to enhance safety monitoring and participant support. Further, the effect of participant medication use on tDCS efficacy is unknown, although it did not seem to affect tDCS tolerability in our study. Finally, stimulus presentation speed on the web-based Sorting SST was variable due to differences in home internet speeds, resulting in variability in the latency of stimulus presentation and RT measurement. Future studies should consider using integrated cloud-based systems for at-home trials and incorporate longer-term follow-up periods.

## 5. Conclusions

We report a safe and feasible method for at-home delivery of tDCS to improve attention in children with ABI, including a detailed discussion on the nuances of conducting at-home tDCS in this population. Further, we also show that HD-EEG may be a useful technique to measure neurophysiological response to non-invasive brain stimulation. This study supports future development of a larger-scale, randomized, double-blinded, placebo-controlled trial to evaluate the efficacy of home-based tDCS to improve attention in children with ABI.

## Figures and Tables

**Table 1:** Baseline demographic, neurocognitive, behavioral, and quality-of-life measures.

	1 mA Group	2 mA Group
Age at baseline (years), mean (95% CI)	11.9 (8.2, 15.4)	13.7 (11.0, 16.5)
Age at injury (years), mean (95% CI)	7.0 (−0.4, 14.5)	9.6 (1.4, 17.8)
Time post-injury (years), mean (95% CI)	4.8 (−0.7, 10.2)	3.9 (−2.2, 10.0)
Sex, *n* males (%)	4 (66.7)	1 (25.0)
Handedness, *n* right (%)	5 (83.3)	4 (100.0)
Special help at school, *n* (%)	4 (66.7)	4 (100.0)
Medication, *n* yes (%)	4 (66.7)	2 (50.0)
TBI severity		
Mild	4 (66.6%)	2 (33.3%)
Severe	2 (33.3%)	2 (33.3%)
CNSVS NCI, mean (95% CI)	91.4 (72.2, 110.6)	100.0 (79.1, 120.9)
CNSVS RT, mean (95% CI)	104.8 (53.1, 156.5)	99.5 (53.6, 145.4)
CNSVS complex attention, mean (95% CI)	74.8 (32.9, 116.7)	104.5 (93.6, 115.4)
BRIEF2 inhibit, mean (95% CI)	65.4 (47.8, 83.0)	59.5 (52.6, 66.4)
BRIEF2 shift, mean (95% CI)	66.6 (45.1, 88.1)	69.5 (40.7, 98.3)
Conners 4 inattention, mean (95% CI)	61.2 (41.2, 81.2)	68.7 (47.7, 89.8)
Conners 4 hyperactivity, mean (95% CI)	62.2 (39.9, 84.5)	57.8 (35.0, 80.5)
Conners 4 impulsivity, mean (95% CI)	65.6 (46.2, 85.0)	59.5 (34.9, 84.1)
Conners 4 ADHD score, mean (SD)	17.2 (12.8)	21.3 (9.9)

Participants analyzed on the basis of dosage received, not assigned. For CNSVS and BRIEF2, standard scores presented. mA, milliamp; SD, standard deviation; CI, confidence interval; TBI, traumatic brain injury; CNSVS, CNS Vital Signs; NCI, neurocognitive index; RT, reaction time; BRIEF2, Behavior Regulation Inventory of Executive Function 2.

## Data Availability

De-identified data presented in this study are available on request from the corresponding author due to the small sample size, which could result in de-identification if made openly available.

## Supplementary Materials

The following supporting information can be downloaded at https://www.mdpi.com/article/10.3390/brainsci15060561/s1, Figure S1: Participant ratings of tDCS sensations for a single participant who could not initially tolerate the full tDCS session; Figure S2: Using whole-brain network-based statistics analysis, several subnetworks showed significantly increased connectivity following home-based tDCS with online attention training. Table S1: Mean RT change following tDCS intervention; Table S2: Mean ‘Fairy Game’ SST performance across intervention; Table S3: Mean ‘Sorting Game’ SST performance across intervention; Table S4: Nodes in NBS subnetworks showing significant differences between pre- and post-tDCS (whole-brain analysis). References [98,99,100,101,102,103,104,105,106,107,108,109,110,111,112,113,114,115,116] are cited in the Supplementary Materials.

## Abbreviations

The following abbreviations are used in this manuscript:

tDCS	Transcranial direct current stimulation
TBI	Traumatic brain injury
HD-EEG	High-density electroencephalography
ABI	Acquired brain injury
ECN	Executive control network
SN	Salience network
DMN	Default mode network
dlPFC	Dorsolateral prefrontal cortex
NBS	Network-based statistics
E-field	Electric field
ADHD	Attention deficit/hyperactivity disorder
ROI	Region of interest
CAT	Computational anatomy toolbox
