# Supplementary material for: Safety, Feasibility, and Tolerability of Ten Days of At-Home, Remotely Supervised tDCS During Gamified Attention Training in Children with Acquired Brain Injury: An Open-Label, Dose-Controlled Pilot Trial
Source: Brain Sci. 2025 May 24;15(6):561. doi: 10.3390/brainsci15060561 (PMC12190726; doi:10.3390/brainsci15060561)
Supplement: Supplementary file 1 [file brainsci-15-00561-s001.zip › brainsci-3631676-supplementary.pdf]

Supplementary Methods

tDCS exclusion criteria

Metal implants, serious or unstable illness or medical condition, current or suspected pregnancy, current lactation, skull defects underneath stimulation sites, scalp wound/skin problem preventing placement of HD-EEG and/or stimulation leads, and epilepsy or other seizure disorders.

Intervention details

The tDCS intervention was given using the NeuroConn DC Stimulator Mobile:

EC declaration number: KE-DCS_DCSM-MDR(Q)-2024-05-27
Certificate: G10 089300 0007 Rev. 00
NB Identification number: CE 0123, document updated 27/05/2024
https://info.neurocaregroup.com/hubfs/neuroCare_May_2021/pdf/EG_Konformit%C3%A4tserkl%C3%A4rung_DC_STIMULATOR.pdf

Gamified attention training

In the 'Fairy Game' SST, the participant was required to find their way out of an enchanted forest by pressing the left or right arrow keys as quickly as possible. Participants were instructed to withhold their responses when an auditory stop signal was presented (representing an 'evil witch'). The 'Fairy Game' SST has been used previously in pediatric ABI[1] and ADHD populations[2] and has been validated against the traditional SST in healthy adults.[3] This game has also been shown to be sensitive to the effects of tDCS targeting the dlPFC.[4] In the 'Sorting Game' SST, items moving down a conveyor belt (animals or food items) were required to be sorted into their appropriate bins as quickly as possible using the left or right arrow keys. Participants were required to withhold their response when sparks flew out of the conveyor belt, representing a 'breakdown' (visual stop signal). The Sorting SST has been validated as a measure of inhibition in an overweight population.[5] Attention training games were performed on a laptop supplied as part of the study equipment. The dominant hand was used press keyboard arrow keys.

MRI acquisition

MRI was used to assess participant anatomy (T1, T2) using a 3T Siemens Prisma scanner with a 64-channel head coil at the Herston Imaging Research Facility (Brisbane, Australia). MRI was used to assess anatomy (T1, T2), structural connectivity (DTI) and perform electric field modelling. For two participants who opted out of the study MRI, the most recent MRI from a previous study/clinical record was used. T1 scans were acquired using MPRAGE, TR 1880.0ms, TE 2.32 ms, voxel size=0.9x0.9x0.9mm, 192 slices. For T2 Flair, T2 SPC FLAIR, the following parameters were used: TR 5000 ms, TE 389 ms, voxel size 0.9 x 0.9 x0.9 mm, 192 slices. Three diffusion weighted imaging

(DWI) scans were collected in short blocks (< 2.5 mins): 8xb=0, 30xb=900 s/mm^2, 30xb=2000 s/mm^2.

Electric field modelling

The normalized E-field is the conventional metric for stimulation strength estimated as the field strength parallel to the skull, where the peak strength is often seen at the midline. Following segmentation in headreco, head models were computed in SimNIBS 4.0.0. where electric field (E-field) models were created.[6] The left dlPFC/right dlPFC montage was modelled according to F3-anode/F4-cathode (10-20 EEG; electrode size:5x5cm; 4 mm thickness). Electrode y-axis pointed towards FC3 and FC4 for F3 and F4 electrodes, respectively. Anodal and cathodal current intensity was 1 mA/-1mA and 2mA/-2mA in the 1 mA and 2 mA conditions, respectively, and default tissue conductivity values were utilized. Mean E-field magnitude (E_norm, v/m) was calculated in a 10 mm spherical radius ROI with a grey matter mask centred at the cortical projections underneath the anodal and cathodal electrodes at the following MNI locations: F3 (-35.5, 49.4, 32.4) and F4 (40.2, 47.6, 32.1)[6-8]. The E-field distribution map was visualised using gmsh.[9]

HD-EEG acquisition

The EEG flanker task was administered through Psychtoolbox-3 (Psychophysics Toolbox Extensions)[10-12] in MATLAB, which allowed recording of participant keyboard responses. A photodiode was also used to record presentation of flanker stimuli, allowing correction for any hardware or software-related delays.

HD-EEG preprocessing

The EEGLAB toolbox v2022.1[13], implemented through MATLAB Version 9.12 (R2022a, The MathWorks Inc, Natick, Massachusetts), was used to pre-process resting eyes closed (EC), resting eyes open (EO) and Go/No-Go task EEG data. First, the EEG data were trimmed to remove the first and last 60 seconds of recording as participants showed greater restlessness during these periods, leaving three minutes of continuous EEG data. Next, we opted to remove 19 peripheral channels using the 'pop_select' function as described in Angelini et al. 2016[14] and Calbi et al. 2019[15] due to poorer signal quality in these peripheral electrodes, leaving a total of 110 channels in analysis. A high-pass filter was applied at 0.5 Hz, the data was down sampled to 250 Hz, and the channels were re-referenced to the average montage using the 'pop_reref' function. Channels were manually identified for rejection and removed using the 'pop_select' function. An average of 8.9 channels were manually removed in the EO condition, 9.0 channels in the EC condition, and 11.9 in the task condition, predominantly from parietal and occipital regions. The data were then re-referenced to average, and independent component analysis (ICA) was conducted using the 'runica (fastica)' function. Independent components (ICs) were labelled using the 'pop_iclabel' function and ICs identified as muscle, eye, heart, line noise, channel noise or 'other' with greater than 50% probability using the 'select_IC' and 'pop_subcomp' function were

removed. The data were manually inspected, and any remaining noisy channels were removed. Finally, rejected channels were spherically interpolated from neighbouring channels using the ‘pop_interp’ function. Bandpass filtering was conducted using a 4th order Butterworth filter between 0.5 and 45 Hz. Lastly, EO and EC data were epoched into 6 segments of 30 s, as small epochs are associated with greater stabilization of network topology.[16] Task data was epoched as 0 to +2000 ms relative to the stimulus. Manual epoch rejection was conducted to remove any remaining noisy epochs, where an average of 2.6 of 196 trials were rejected. Epochs were then averaged across time for connectivity analysis.

Region of interest definitions

DMN nodes were defined according to the Desikan Killiany atlas bilateral ‘isthmus cingulate’, ‘posterior cingulate’, ‘precuneus’, ‘rostral anterior cingulate’, ‘parahippocampal’ and ‘rostral middle frontal’ nodes.[17] SN nodes were defined as the bilateral ‘caudal anterior cingulate’ and ‘insula’ nodes.[18,19] ECN nodes were defined as the left ‘middle temporal’, bilateral ‘superior parietal’, bilateral ‘caudal middle frontal’, and right caudal anterior cingulate’.[20]

Statistical Analysis

Reaction time

Flanker trials were excluded if RT was <100ms or the response was incorrect.[21] SSRT was calculated using the Verbruggen method.[22] Trials were excluded if the accuracy rate was 75%, if RT on unsuccessful stop trials > RT on go trials (race model violation), or if probability of responding on a stop signal was <0.25 or >0.75.[23] Non-normal RT data was not transformed as previously described.[24]

Supplementary Results

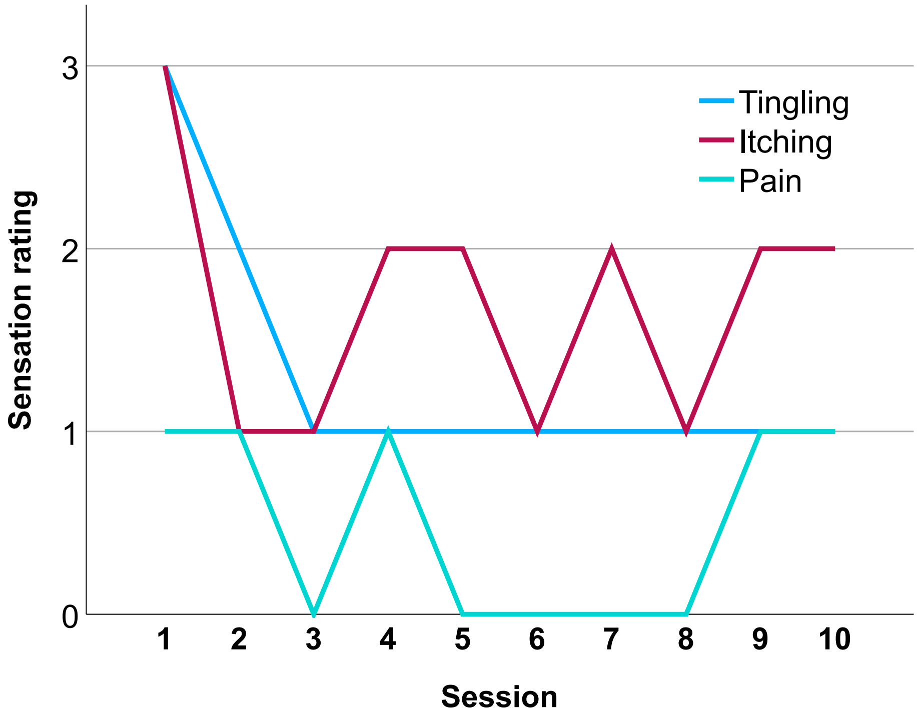

Supplementary Figure 1: Participant rating of tDCS sensations for single participant who could not initially tolerate full tDCS session. tDCS sessions began with five minute duration, increasing duration each day, where the full 20 minute treatment protocol was only completed on Sessions 7-10. Tingling (A), itching (B), and pain (C) sensations during tDCS sessions. Overall sensation rating decrease seen from Session 2 to Session 3. Tolerability questionnaire was not completed by participant on Session 1.

RT change

Supplementary Table 1: Mean RT change following tDCS intervention

	**Flanker RT change (ms, SD) n=9**	**Go RT change (ms, SD) n=8**
**1 mA**	-105.78 (72.47)	67.06 (61.65)
**2 mA**	-61.98 (58.44)	-34.77 (81.63)

mA, milliamps; tDCS, transcranial direct current stimulation; RT, reaction time; ms, milliseconds

Cognitive training tasks during tDCS

Supplementary Table 2: Mean 'Fairy Game' SST performance across intervention

**Measure**	**1 mA group**	**2 mA group**
SSRT (ms), mean (SD)	680.2 (255.6)	539.1 (145.3)
Correct go RT (ms), mean (SD)	1122.1 (47.6)	980.0 (79.8)
Go error rate, mean (SD)	0.150 (0.087)	0.063 (0.048)

SSRT, stop signal reaction time; ms, milliseconds; SD, standard deviation; RT, reaction time; mA, milliamps

Supplementary Table 3: Mean 'Sorting Game' SST performance across intervention

Measure	1 mA group	2 mA group
SSRT (ms), mean (SD)	431.4 (173.5)	393.0 (77.9)
Correct go RT (ms), mean (SD)	1062.9 (258.6)	1045.3 (137.8)
Go error rate, mean (SD)	0.041 (0.036)	0.026 (0.20)

SSRT, stop signal reaction time; ms, milliseconds; SD, standard deviation; RT, reaction time; mA, milliamps

Supplementary Table 4: Nodes in NBS subnetworks showing significant differences between pre and post tDCS (whole brain analysis)

EEG condition	Power spectral band	Direction of connectivity comparison	Nodes in subnetworks	T-statistic threshold	p val
EC	Gamma	Increased following tDCS	Left postcentral<br>Left rostral middle frontal<br>Right cuneus<br>Left temporal pole	3.1	<0.001
EO	Gamma	Increased following tDCS	Left rostral anterior cingulate<br>Right rostral anterior cingulate<br>Left middle temporal<br>Left medial orbitofrontal	3.1	0.02
EO	Beta	Increased following tDCS	Left caudal anterior cingulate<br>Right caudal anterior cingulate<br>Right pars orbitalis<br>Left lateral orbitofrontal<br>Left insula	3.1	0.02
EC	Alpha	Increased following tDCS	Left frontal pole<br>Left fusiform<br>Left rostral anterior cingulate<br>Left superior frontal<br>Right lateral orbitofrontal<br>Right temporal pole	3.1	0.02
EO	Theta	Increased following tDCS	Left lateral occipital<br>Right isthmus cingulate<br>Left caudal middle frontal<br>Left pericalcarine<br>Left lingual<br>Left precuneus<br>Right precentral	3.1	0.01

EC, eyes closed; EO, eyes open; tDCS, transcranial direct current stimulation.

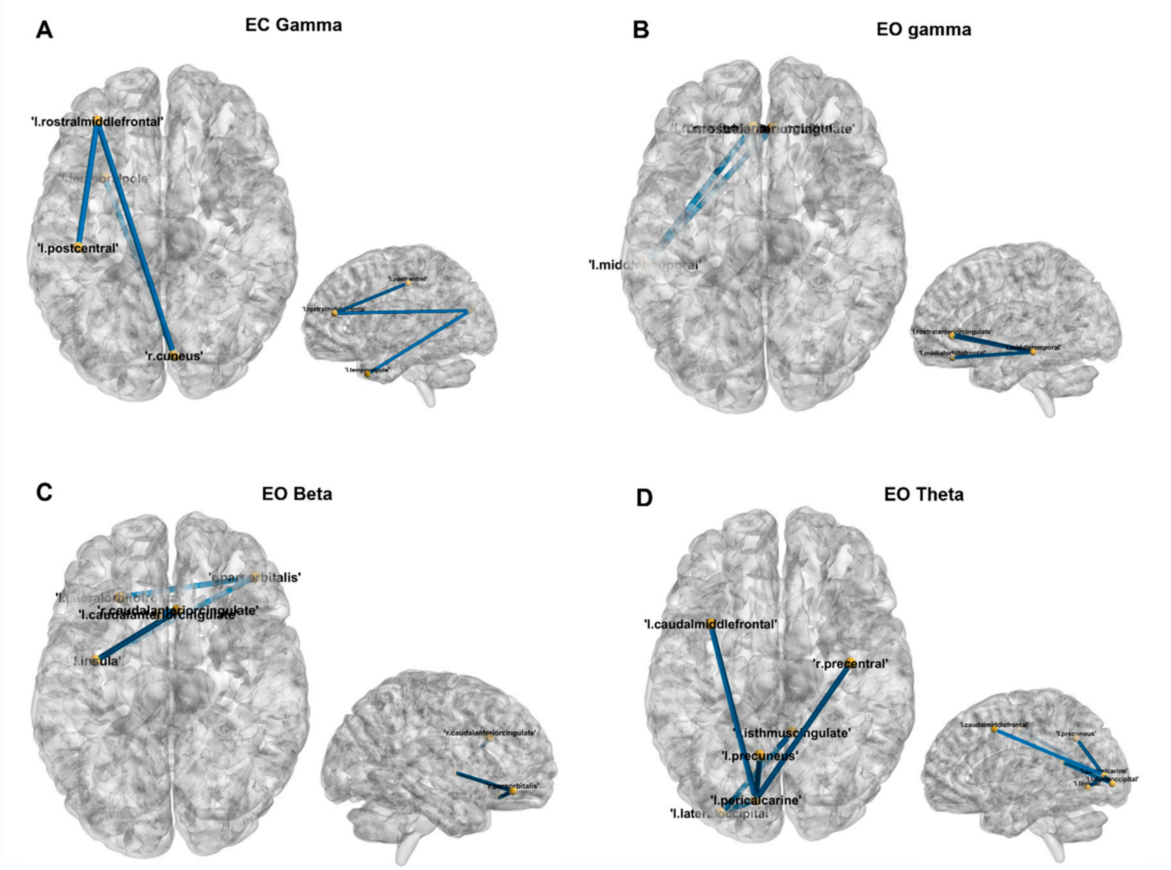

Supplementary Figure 2: Using whole-brain Network-Based Statistics analysis, several subnetworks showed significantly increased connectivity following home-based tDCS with online attention training. Significantly increased fc post-intervention in (A) Gamma band, eyes closed; (B) Gamma band, eyes open; (C) Beta band, eyes closed; (D) Theta band, eyes open. Left side of brain map indicates left side of brain, n=9. BrainNet Viewer used to visualise subnetworks.
EO, resting eyes open; EC, resting eyes closed.

References

1. Stein A, Caulfield KA, Singh M, et al. The effect of a single session of tDCS on attention in pediatric acquired brain injury: Characterising inter-individual structural and functional network response variability. *medRxiv* 2025: 2025.2002. 2027.25323049.
2. Gallagher R, Kessler K, Bramham J, et al. A proof-of-concept study exploring the effects of impulsivity on a gamified version of the stop-signal task in children. *Frontiers in psychology* 2023; 14: 1068229.
3. Friehs MA, Dechant M, Vedress S, et al. Effective gamification of the stop-signal task: two controlled laboratory experiments. *JMIR Serious Games* 2020; 8: e17810.
4. Friehs MA, Dechant M, Vedress S, et al. Shocking advantage! Improving digital game performance using non-invasive brain stimulation. *International Journal of Human-Computer Studies* 2021; 148: 102582.
5. Schroeder PA, Lohmann J and Ninaus M. Preserved Inhibitory Control Deficits of Overweight Participants in a Gamified Stop-Signal Task: Experimental Study of Validity. *JMIR Serious Games* 2021; 9: e25063.
6. Saturnino GB, Puonti O, Nielsen JD, et al. SimNIBS 2.1: a comprehensive pipeline for individualized electric field modelling for transcranial brain stimulation. *Brain Human Body Modeling* 2019: 3-25.
7. Okamoto M, Dan H, Sakamoto K, et al. Three-dimensional probabilistic anatomical cranio-cerebral correlation via the international 10–20 system oriented for transcranial functional brain mapping. *Neuroimage* 2004; 21: 99-111.
8. Caulfield KA and George MS. Optimized APPS-tDCS electrode position, size, and distance doubles the on-target stimulation magnitude in 3000 electric field models. *Scientific Reports* 2022; 12: 1-15.
9. Geuzaine C and Remacle JF. Gmsh: A 3-D finite element mesh generator with built-in pre- and post-processing facilities. *International journal for numerical methods in engineering* 2009; 79: 1309-1331.
10. Brainard DH and Vision S. The psychophysics toolbox. *Spatial vision* 1997; 10: 433-436.
11. Pelli DG and Vision S. The VideoToolbox software for visual psychophysics: Transforming numbers into movies. *Spatial vision* 1997; 10: 437-442.
12. Kleiner M, Brainard D and Pelli D. What's new in Psychtoolbox-3? 2007.
13. Delorme A and Makeig S. EEGLAB: an open source toolbox for analysis of single-trial EEG dynamics including independent component analysis. *Journal of Neuroscience Methods* 2004; 134: 9-21.
14. Angelini M, Calbi M, Ferrari A, et al. Proactive control strategies for overt and covert go/nogo tasks: an electrical neuroimaging study. *PloS one* 2016; 11: e0152188.
15. Calbi M, Siri F, Heimann K, et al. How context influences the interpretation of facial expressions: a source localization high-density EEG study on the “Kuleshov effect”. *Scientific reports* 2019; 9: 2107.
16. Fraschini M, Demuru M, Crobe A, et al. The effect of epoch length on estimated EEG functional connectivity and brain network organisation. *Journal of neural engineering* 2016; 13: 036015.
17. Roig-Herrero A, Planchuelo-Gómez Á, Hernández-García M, et al. Default mode network components and its relationship with anomalous self-experiences in schizophrenia: A rs-fMRI exploratory study. *Psychiatry Research: Neuroimaging* 2022; 324: 111495.
18. Pimontel MA, Solomonov N, Oberlin L, et al. Cortical thickness of the salience network and change in apathy following antidepressant treatment for late-life depression. *The American Journal of Geriatric Psychiatry* 2021; 29: 241-248.
19. Metzler-Baddeley C, Caeyenberghs K, Foley S and Jones DK. Task complexity and location specific changes of cortical thickness in executive and salience networks after working memory training. *Neuroimage* 2016; 130: 48-62.
20. Shen Kk, Welton T, Lyon M, et al. Structural core of the executive control network: A high angular resolution diffusion MRI study. *Human brain mapping* 2020; 41: 1226-1236.
21. Whelan R. Effective analysis of reaction time data. *The psychological record* 2008; 58: 475-

482.
22. Verbruggen F and Logan GD. Models of response inhibition in the stop-signal and stop-change paradigms. *Neuroscience and Biobehavioral Reviews* 2009; 33: 647-661.
23. Verbruggen F, Aron AR, Band GP, et al. A consensus guide to capturing the ability to inhibit actions and impulsive behaviors in the stop-signal task. *elife* 2019; 8: e46323.
24. Lo S and Andrews S. To transform or not to transform: Using generalized linear mixed models to analyse reaction time data. *Frontiers in psychology* 2015; 6: 1171.
